# Non drug-related and opioid-specific causes of 3262 deaths in Scotland’s methadone-prescription clients, 2009–2015

**DOI:** 10.1016/j.drugalcdep.2019.01.019

**Published:** 2019-04-01

**Authors:** Lu Gao, J. Roy Robertson, Sheila M. Bird

**Affiliations:** a MRC Biostatistics Unit, University of Cambridge School of Clinical Medicine, Cambridge CB2 0SR, United Kingdom; b University of Edinburgh Usher Institute, Edinburgh EH8 9AG, United Kingdom; c University of Edinburgh Centre for Medical Informatics, Edinburgh EH16 4UX, United Kingdom

**Keywords:** Methadone clients, Drugs-related deaths, Methadone-specific deaths, Age-group, Co-present circulatory, respiratory or digestive disease, Quantity of prescribed methadone, Interaction between quantity and age-group, Age-related predominance of non drug-related deaths

## Abstract

**Background:**

Opioid drug use is a major cause of premature mortality, with opioid substitution therapy the leading intervention. As methadone-clients age, non-drug-related deaths (non-DRDs) predominate and DRD-risks increase differentially, quadrupling at 45+ years for methadone-specific DRDs.

**Methods:**

36,606 methadone-prescription-clients in Scotland during 2009–2015 were linked to mortality records to end-2015 by their Community Health Index (CHI). Cohort-entry, also baseline quantity of prescribed methadone, were defined by clients’ first CHI-identified methadone-prescription during 2009–2015. National Records of Scotland identified non-DRDs from DRDs; and provided ICD10 codes for underlying and co-present causes of death. Methadone-specific DRD means methadone was implicated in DRD but neither heroin nor buprenorphine.

**Results:**

During 193,800 person-years of follow-up, 1939 non-DRDs (59%) and 1323 DRDs occurred, of which 546 were methadone-specific. Predominant underlying ICD10 chapters for non-DRDs were: neoplasm (377); external causes (341); diseases of digestive (303), circulatory (286) or respiratory (212) system. As methadone-clients aged, the non-DRD proportion of their deaths increased from 54% (717/1318) at 35–44 years to 89% (372/417) at 55+ years.

After allowing for DRDs’ opioid-specificity, age-group and quintile for last-prescribed methadone, there was a significant, positive interaction for co-present circulatory disease between top-quintile for prescribed methadone and 45+ years at death (p = 0.033 after Bonferroni); not for digestive or respiratory co-presence.

**Conclusions:**

Circulatory disease is the co-morbidity most likely implicated in the quadrupling of methadone-specific DRD-risk at 45+ years; followed by digestive disease. Cultural shift is needed in treatment-services because degenerative non-DRDs predominate as methadone-clients age. Future linkage-studies should access hospitalizations and methadone-daily-dose.

## Highlights

• 36,606 methadone-prescription clients in Scotland had 193,800 person=-years of follow-up during 2009-15.
• Clients experienced 3262 deaths: 1939 non-DRDs (59%) and 1312 DRDs of which 546v were methadone-specific.
• The non-DRD proportion increased from 54% [717/1318] at 35-44 years through 65% [589/907] at 45-54 to 89% [372/417] at 55+ years.
• Predominant underlying non-DRD causes were neoplasms (377); external causes (341); digestive (303), circulatory (286) or respiratory (212) disease.
• Only co=present circulatory disease showed a significant interaction between older age and the top quintile for quantity of methadone prescribed.

## 1 Introduction

For Scotland, we planned to document ageing methadone-clients’ non drug-related deaths (non-DRDs) and investigate which of three major co-morbidities (circulatory, digestive, respiratory) might be implicated in clients’ steep rise in methadone-specific DRDs with age.

*Scene-setting:* In Europe and North America, the majority of DRDs is opioid-related. Survivors of the heroin-injector epidemics of the late 1970s and early 1980s, now in their 40 s and 50 s, mostly receive opioid substitution therapy which, in the UK, is methadone predominantly.

North America’s dramatic recent rise in opioid-related DRDs has been exacerbated by fentanyl; but also by the over-prescription of opiates for pain management which is an additional reason for opioid-related DRDs there to be ageing.

There is strong evidence that opioid users’ DRD-risk increases with age; and that younger females are at much lower DRD-risk than young males, an advantage which diminishes with age. In the UK, evidence of these demographic influences is consistent across a) evidence-syntheses, b) cohort studies and c) official statistics (Bird et al., 2010; Merrall et al. (2012); King et al. (2014, 2013); Pierce et al. (2015a, 2015b); White et al., 2015; Gao et al., 2016; National Records of Scotland (NRS), 2018).

More recently, Gao et al. (2016) and Pierce et al. (2018) showed that the risk of methadone-specific DRD (that is: methadone implicated in DRD but neither heroin nor buprenorphine) rises steeply with age, quadrupling at 45+ years of age compared with 25–34 years. They speculated that circulatory, digestive or respiratory co-morbidities might be implicated.

*Only cohort studies reveal non-DRD mortality and morbidity rates:* Uncounted by official statistics is the predominance of non-DRDs as opioid users age. People who use drugs have a range of physical co-morbidities, which may potentiate opioid-related DRD-risks and are themselves major causes of non-DRDs. Important among them are diseases of the circulatory, gastrointestinal (including liver) and respiratory systems (Degenhardt et al., 2009; Merrall et al., 2013; Merrall ELC, 2012; Pierce et al., 2015b).

Smoking is a major risk-factor for both circulatory and respiratory disease while hepatitis viruses, alcohol misuse and obesity all herald liver disease. Half of Scotland’s prisoners with a history of injection drug use are Hepatitis C virus (HCV) carriers, as revealed in prison studies from 1994 to 2010 (Gore et al., 1999; Taylor et al., 2013).

Diagnosis of HCV, older age (35+ years) and declared misuse of alcohol were the top three predictors for Scottish Drugs Misuse Database clients’ risk of death from digestive system disease in 1996–2006 (Merrall ELC, 2012). Cause-specific mortality in 2005–2009 for over 150,000 opiate-dependent clients in England’s National Drug Treatment Monitoring System revealed two underlying causes which accounted for at least 10% of client-deaths, namely: circulatory (418 cases) and digestive system disease (423 cases). The same two topped Scottish clients’ list of hospital-episode-rates by discharge-diagnosis: 25 hospital-episodes per 1000 person-years (digestive), 18 (circulatory), with 14 per 1000 person-years for respiratory disease (Merrall et al., 2013; Merrall ELC, 2012).

*Opioid-specificity of DRDs, as documented by official statistics:* Scotland’s methadone-related deaths have been a major concern since 2011 when, for the first time, they outnumbered Scotland’s heroin-related deaths (Ferguson, 2012; Scottish Drug Strategy Delivery Commission, 2013; Strang et al., 2010). Near equality occurred in 2012, 2013 and again in 2017 (National Records of Scotland, 2018).

Hence, Gao et al. (2016) investigated the opioid-specificity of DRDs experienced by Scotland’s methadone-prescription-clients in 2009–2013. Their key finding was that the hazard-rate for methadone-specific DRD increased steeply with age-group, doubling at 35–44 years and nearly quadrupled at 45+ years (versus 25–34 years), both in Scotland and England (Gao et al., 2016; Pierce et al., 2018).

But underlying mechanisms are not well understood (Hickman et al., 2018), with the following potentially implicated: co-morbid circulatory, digestive or respiratory disease; quantity of prescribed methadone; altered half-life of methadone by gender, age-group or liver disease; and co-prescribing for mental ill-health (Gao et al., 2016; Pierce et al., 2018).

*Capitalizing on co-morbidities determined at forensic autopsy:* Understanding whether ageing methadone-clients’ pre-existing co-morbid conditions influence the opioid-specificity of their DRDs is now critical. Since DRDs are subject to forensic autopsy, co-morbid conditions recorded for DRDs have the advantage of histopathological assessment. In addition, a national protocol for toxicology at forensic autopsy in Scotland underpins the opioid-specificity of Scotland’s DRDs.

In this paper, we revisit Scotland’s methadone-prescription-client cohort, this time for 2009–2015, first to elucidate the increasingly-aged clients’ major causes of non-DRDs and secondly to clarify how three major co-morbidities (circulatory, digestive, respiratory) relate to the opioid-specificity of DRDs, prescribed methadone and age-group.

Both objectives will be addressed by analysing how major chapters of the International Classification of Diseases (10th edition, ICD10) account for underlying and co-present causes of death for Scotland’s 2009–2015 methadone-prescription-client cohort. We estimated that linkage to mortality records to 31 December 2015 would yield around 3200 deaths, including 1200 DRDs (half of them methadone-specific); and that several ICD10 chapters, including for circulatory, digestive and respiratory diseases, would be underlying for 250–350 deaths but co-present for up to twice as many. The quantity of prescribed-methadone was available for each prescription.

The present study aims to do the following:

i) highlight major ICD10-chapters for the underlying cause of ageing methadone-prescription-clients’ death when death is non-DRD;
ii) identify whether the ICD10-chapters for circulatory, digestive and respiratory disease are differentially co-present at opioid-specific DRDs (especially at 45+ years of age);
iii) summarize quantity of prescribed methadone for opioid-specific DRDs and for non-DRDs (especially at 45+ years of age);
iv) derive logistic risk-scores for co-presence of circulatory, digestive or respiratory disease, which take into account opioid-specificity, age-group at DRD and quintile for quantity of prescribed methadone;
v) within each risk-score, test for a significant positive interaction between top quintile of prescribed methadone and 45+ years of age.

## 2 Methods

### 2.1 Definitions: Drug-related Deaths; and ICD10 Coding for all Deaths

We used the UK harmonized definition of DRD (National Records of Scotland, 2017) and requested information on the opioid-specificity of Scotland’s DRDs from NRS as follows:

**methadone-specific DRDs**: methadone was implicated in DRD but neither heroin/morphine nor buprenorphine implicated;

**heroin-specific DRDs**: heroin/morphine was implicated in DRD but neither methadone nor buprenorphine implicated; and

**heroin-methadone DRDs**: methadone and heroin/morphine both implicated but buprenorphine was not implicated.

In appraising which drugs are implicated as causal factors in any DRD and which, although present, probably did not contribute, Scotland’s pathologists are supported by having a national protocol for toxicological testing at forensic autopsies.

For each death, in addition to underlying cause of death, NRS also provided all mentioned ICD10 sub-codes.

### 2.2 Scotland’s Community Health Index (CHI)

Scotland’s CHI is a register of all patients in NHS Scotland, Scotland’s publicly funded healthcare system. Patients are identified by a 10-digit CHI-number, usually the patient’s date of birth (DDMMYY) followed by four digits: two randomly generated, the third identifying gender (odd for males), and the fourth a check-digit. See Pavis and Morris (2015) on the importance of CHI-numbers for Scotland’s trusted record-linkage.

### 2.3 Scotland’s Methadone-prescription-client Cohort for 2009–2015: Data-sources and Linkage

Methadone prescriptions are held within the Scottish National Prescribing Information System (Alvarez-Madrazo et al., 2016): most are CHI-identified and all give quantity of methadone prescribed. Neither daily-dose of prescribed methadone nor the prescription’s duration is routinely available electronically as neither is needed for reimbursing pharmacists.

To define Scotland’s methadone-prescription-client cohort for 2009–2015, nearly 3 million methadone prescriptions during 1 July 2009 to 30 June 2015 had to be linked to NRS’s mortality records to 31 December 2015. Because all deaths are CHI-identified, Scotland’s CHI-number (if available per-prescription) was used for this exact linkage. The CHI-number was also used to link serial CHI-identified methadone-prescriptions for the same client.

For CHI-indexed methadone-prescriptions, we obtained data as follows: client’s gender and age in completed years at 1 July of prescription-year, enabling analysis by age-group at death (coded: <35 years, 35–44, 45–54, 55+ years); prescription-date (when missing, the later re-imbursement date was used); re-imbursement-date; quantity of prescribed methadone per prescription; daily-dose of prescribed methadone and duration of prescription (if extractable from electronic messaging using Natural Language Programs at Information Services Division); full date of death; ICD10 codes for underlying and co-present causes of death; whether the underlying (ie main) cause of death was DRD or non-DRD.

As full date of death is potentially identifying, computations were conducted via the Farr Institute’s safe-haven in Scotland.

### 2.4 Inclusion/Exclusion Criteria

We defined Scotland’s methadone-prescription-client cohort for 2009–2015 as: clients who had received one or more CHI-identified methadone prescription (from general practitioner (GP) or other sources) during 1 July 2009 to 30 June 2015. Client’s date of accrual was date of the client’s first CHI-identified methadone prescription during 1 July 2009 to 30 June 2015. That prescription also defined the client’s baseline quantity of prescribed methadone and age-group at accrual (<25 years, 25–34, 35–44, 45+ years).

Initial check on the integrity of date-sequences was made by computing the time interval in days from the date of the client’s baseline CHI-identified methadone prescription to the earlier of death-date and 31 December 2015. Sixty-three negative survival times were referred back to Information Services Division for cross-checking, only seven of which were considered to be incorrectly linked; and have been deleted. As in Gao et al. (2016), we otherwise added a fixed number of days (here 60 days) to all survival intervals to ensure positivity.

### 2.5 Key Variables

The opioid-specificity of DRDs, defined above, was a primary consideration.

Age-group at accrual (<25 years, 25–34, 35–44, 45+ years) was defined for consistency with Gao et al. (2016) but age-group at subsequent death was more appropriately defined as: <35 years, 35–44, 45–54, 55+ years.

Quantity of prescribed methadone (either at baseline or according to the latest CHI-identified prescription in the year prior to death) was summarized by mean (sd) and inter-quartile range; or quintiles.

In logistic regression for the odds on co-present circulatory, digestive or respiratory disease, quantity prescribed was analysed by quintiles as in Gao et al. (2016) but abbreviated to bottom, mid, top quintiles and the quantity-age interaction to be tested was defined by: top quintile for quantity of prescribed-methadone (based on the latest CHI-identified prescription in the year prior to death) and aged 45+ years at death.

### 2.6 Statistical Analysis

We first documented underlying cause of death by ICD10 chapter; then ICD10 chapters of which there was any-mention in the cause of death for Scotland’s methadone-prescription cohort in 2009–2015.

Next, we assessed whether the age-related pattern of co-present mentions of specific ICD10-chapters was different by DRDs’ opioid-specificity, notably for circulatory, digestive or respiratory disease and methadone-specific DRDs. We added undetermined intent (Y-codes) post hoc. Differential patterns at 45+ years of age could potentially shed light on the co-morbidities that might account for the steeply increased hazard ratios by age for methadone-specific DRD, as previously documented by Gao et al. (2016) and Pierce et al. (2018).

By age-group, we also compared the mean quantity of prescribed methadone at baseline (and at latest CHI-identified prescription in the year prior to death) for those whose subsequent death was methadone-specific DRD or otherwise.

Finally, we derived logistic regression-scores for the co-presence at opioid-specific DRDs of circulatory; digestive; respiratory disease (prior hypotheses); or undetermined intent (Y-code). Besides opioid-specificity, age-group at death and quintile for latest quantity of prescribed methadone in the deceased’s final-year were fitted; and an interaction between top quintile for prescribed methadone and age 45+ years at opioid-specific DRD was tested for. The reported interaction p-value should be multiplied by 3, as a Bonferroni correction for having had three prior hypotheses.

For translating from odds [O] on co-present circulatory disease to estimated probability [P] of co-present circulatory disease, use: P = O/[1 + O].

## 3 Results

### 3.1 Scotland’s 2009–2015 Methadone-prescription-client Cohort

During 1 July 2009 to 30 June 2015, Scotland had 2,922,052 methadone prescriptions. As in Gao et al. (2016), the percentage of CHI-identified methadone prescriptions was **66%** (1,932,090/2,922,052) but the estimated proportion of methadone-prescription-clients with at least one CHI-identified prescription in 2009–2015 was **80%** (36,606/46,000 to nearest 100).

The majority (57%, 20,757 clients) joined the CHI-identified cohort of methadone-prescription-clients during July to December 2009, being mostly prevalent clients, see Table 1. During 193,813 person-years of follow-up to 31 December 2015, there were 3262 deaths. The cohort experienced 1939 non-DRDs (59%) and 1323 DRDs: rates of 10.0 non-DRDs (95% CI: 9.5–10.5) and 6.8 DRDs (95% CI: 6.4–7.2) per 1000 pys respectively.

**Table 1:** Baseline characteristics for Scotland’s 36,606 CHI-indexed methadone-prescription clients, 2009–2015.

**Covariates defined by** **1^st^ CHI-identified methadone prescription during** **1 July 2009 to 30 June 2015**		**Clients** **N = 36,606**	**Non-DRDs** **N =** 1,939	**DRDs** **N =** 1,323	**PERSON-YEARS (%): mean person-years (pys) per client** **PYS = 193,813: 5.3 pys**
**Cohort-entry period**	July – Dec 2009	20,757 (57)	1,220 (63)	857 (65)	129,896 (67): 6.3 pys
	2010	6,534 (18)	348 (18)	256 (19)	35,409 (18): 5.4 pys
	2011	3,034 (8)	153 (8)	111 (8)	13,469 (7): 4.4 pys
	2012	1,965 (5)	74 (4)	45 (3)	6,952 (4): 3.5 pys
	2013	1,857 (5)	72 (4)	29 (2)	4,694 (2): 2.5 pys
	2014	1,704 (5)	56 (3)	23 (2)	2,731 (1): 1.6 pys
	January – June 2015	755 (2)	16 (1)	2 (nil)	663 (< 1): 0.9 pys
**Prescription source**	GP-prescription	23,760 (65)	1,426 (74)	847 (64)	131,379 (68): 5.5 pys
	Other-source	12,846 (35)	513 (26)	476 (36)	62,434 (32): 4.9 pys
**Gender**	Males	24,369 (67)	1350 (70)	908 (69)	128,492 (66): 5.3 pys
	Females	12,237 (33)	589 (30)	415 (31)	65,322 (34): 5.3 pys
**Age-group at cohort-entry**	Under 25 years	2,899 (8)	49 (3)	61 (5)	14,366 (7): 5.0 pys
	25 – 34	15,953 (44)	404 (21)	503 (38)	86,812 (45): 5.4 pys
	35 – 44	13,498 (37)	804 (41)	565 (43)	72,768 (38): 5.4 pys
	45 + years	4,256 (12)	682 (35)	194 (15)	19,868 (10): 4.7 pys
**QUINTILE for baseline quantity of prescribed methadone**	Q1: 1–240 mg	7,462	412	223	33,355 (17): 4.5 pys
	Q2: 241–616 mg	7,187	406	273	36,654 (19): 4.6 pys
	Q3: 617–1120 mg	7,674	373	265	42,553 (22):5.6 pys
	Q4: 1121–1960 mg	7,804	413	280	43,656 (23): 5.6 pys
	Q5: > = 1961 mg	7,479	335	282	37,595 (19): 5.0 pys

**QUINTILE for available baseline daily-dose of prescribed methadone**	**Baseline daily-dose not available**	**26,226 (72%)**	1,392 **(72%)**	**979 (74%)**	**132,966 pys (69%): 5.1 pys**
	Q1: 1 - 30 mg	2,350	173	72	13,008 (7): 5.5 pys
	Q2: 31 - 50 mg	2,206	104	67	12,965 (7): 5.9 pys
	Q3: 51 - 70 mg	2,182	113	71	13,006 (7): 6.0 pys
	Q4: 71 - 90 mg	1,844	76	66	11,066 (6): 6.0 pys
	Q5: 91 – 280 mg	1,798	81	68	10,802 (6): 6.0 pys

**Boldface**: for headings.

The GP-subset (44%) of 10,380 methadone-prescription-clients for whom baseline daily dose of prescribed methadone was available electronically had significantly lower rates of non-DRDs (9.0; 95% CI: 8.3–9.8) and DRDs (5.7; 95% CI: 5.1–6.3) per 1000 pys than all other clients for whom daily dose was not available (non-DRDs 10.5; 9.9–11.0 and DRDs 7.4; 95% CI: 6.9–7.8).

### 3.2 Underlying Cause of Death by Age-group at Death

Of all 3262 deaths, 1323 (41%) were DRDs, of which 1175 (89%) were opioid-specific: 546 methadone-specific; 320 heroin-specific; both heroin and methadone (but not buprenorphine) implicated in 309 DRDs. Buprenorphine was implicated in 27 DRDs. There were 121 DRDs in which none of heroin/morphine, methadone or buprenorphine was implicated.

Five underlying or main causes of death, each responsible for at least 10% of 1939 non-DRDs, together accounted for 75% (1457) of methadone-prescription-clients’ non-DRDs: neoplasms (377, 21%, of which 113 were malignant neoplasm of unspecified part of bronchus or lung); external causes of morbidity and mortality (primarily accidents, other causes of accidental injury, intentional self-harm, assault, events of undetermined intent, ICD10 code-letters X: 279, 14%; Y: 62, 3%); diseases of the circulatory (286, 15%), digestive (303, 16%) or respiratory system (212, 11%).

Underlying causes for non-DRDs differed by age-group with neoplasms, circulatory, digestive and respiratory system diseases being the equal-top main causes at 45–54 years (when they accounted for 66% of non-DRDs) whereas neoplasms alone accounted for 48% of methadone-clients’ non-DRDs at 55+ years, with circulatory and respiratory causes being joint-second at 16% each, see Table 2.

**Table 2:** Underlying (or main) ICD10 chapter for all 1939 non-DRDs; by age-group at death.

ICD10 codes	ICD10 chapter, see http://apps.who.int/ classifications/icd10/browse/2010/en	Age-at-death frequency of ICD10 chapter for underlying causes of methadone-prescription-clients’ non-DRDs				
		Frequency (%) in 1939 non-DRDs	Frequency (%) in 261 clients aged < 35 years at death	Frequency (%) in 717 clients aged 35-44 years at death	Frequency (%) in 589 clients aged 45-54 years at death	Frequency (%) in 372 clients aged 55+ years at death
A	Certain infectious and parasitic diseases	26 (1.3)	5 (1.9)	10 (1.4)	9 (1.5)	2 (0.5)
B		83 (4.3)	1 (0.4)	34 (4.7)	37 (6.3)	11 (3.0)
C	**Neoplasms**	**377 (19.4)**	14 (5.4)	**78 (10.9)**	**108 (18.3)**	**177 (47.6)**
E	Endocrine, nutritional and metabolic diseases	35 (1.8)	8 (3.1)	16 (2.2)	9 (1.5)	2 (0.5)
F	**Mental and behavioural disorders**	**120 (6.2)**	**30 (11.5)**	48 (6.7)	34 (5.8)	8 (2.2)
G	Diseases of nervous system	26 (1.3)	2 (0.8)	8 (1.1)	12 (2.0)	4 (1.1)
I	**Diseases of circulatory system**	**286 (14.8)**	20 (7.7)	**114 (15.9)**	**93 (15.8)**	**59 (15.9)**
J	**Diseases of respiratory system**	**212 (10.9)**	7 (2.7)	63 (8.8)	**85 (14.4)**	**57 (15.3)**
K	**Diseases of the digestive system**	**303 (15.6)**	**34 (13.0)**	**137 (19.1)**	**104 (17.7)**	28 (7.5)
R	Symptoms, signs and abnormal clinical and laboratory findings, not elsewhere classified	37 (1.9)	6 (2.3)	16 (2.2)	12 (2.0)	3 (0.8)
X	**External causes of morbidity and mortality**	**279 (14.4)**	**91 (34.9)**	**131 (18.3)**	47 (8.0)	10 (2.7)
Y		62 (3.2)	20 (7.7)	28 (3.9)	14 (2.4)	Nil

*Selected sub-codes: respiratory; digestive system; external causes*						
*J09-18*	*Influenza and pneumonia*	53 (2.7)	4 (1.5)	22 (3.1)	19 (3.2)	8 (2.2)
*J40-47*	*Chronic lower respiratory disease*	134 (6.9)	3 (1.2)	35 (4.9)	55 (9.3)	41 (11.0)

*K70-77*	*Liver disease*	265 (13.7)	29 (11.1)	123 (17.2)	94 (16.0)	19 (5.1)
*K70*	*Alcoholic liver disease*	213 (11.0)	25 (9.6)	102 (14.2)	75 (12.7)	11 (3.0)
*K74*	*Fibrosis and cirrhosis*	31 (1.6)	3 (1.2)	12 (1.7)	12 (2.0)	4 (1.1)

*X65-84* *Y15-34*	*Non-DRD suicide*	191 (9.9)	63 (24.1)	95 (13.2)	32 (5.4)	1 (0.3)
*X86-Y09*	*Homicide*	56 (2.9)	23 (8.8)	21 (2.9)	8 (1.4)	4 (1.1)

**Boldface:** for underlying ICD10 chapters which accounted for more than 5% of 1939 non-DRDs; and, for them, to highlight significantly differential frequency by age-group.

Around half (54%, 717) of 1318 deaths aged 35–44 years were non-DRDs, rising to 65% at 45–54 years (589/907) and 89% (372/417) at 55+ years.

### 3.3 Co-present ICD10 Codes by Age-group at Death and Opioid-specificity of DRD

For co-present circulatory, digestive or respiratory system disease and for ICD10 code-letters X and Y (undetermined intent), Table 3 gives the age-group at death for non-DRDs versus for opioid-specific DRDs; and for all 3262 deaths.

**Table 3:** Age-group at death for non-DRDs versus for opioid-specific DRDs – for selected co-present ICD10 chapters, including Y for undetermined intent. **Boldface** for ICD10 chapters and Totals.

Age-group at death (%)	Sub-grouping of all deaths				Total of 3262 deaths
	1939 Non-DRDs	546 Methadone-specific DRDs	320 Heroin-specific DRDs	309 Methadone + heroin DRDs	
< 35 years	261 (13.5)	123 (22.5)	107 (33.4)	96 (31.1)	620 (19.0)
35-44 years	717 (37.0)	236 (43.2)	155 (48.4)	135 (43.7)	1318 (40.4)
45-54 years	589 (30.1)	165 (30.2)	53 (16.6)	70 (22.7)	907 (27.8)
55+ years	372 (19.2)	22 (4.0)	5 (1.6)	8 (2.6)	417 (12.8)
**Totals**	**1939**	**546**	**320**	**309**	**3262**
Co-present ICD10-code frequencies [relative frequency as % of the above age- and sub-group specific deaths]					

**ICD10 code-I: diseases of the circulatory system**					
< 35 years	41 [16%]	7 [6%]	4 [4%]	3 [3%]	59 [10%]
35-44 years	190 [27%]	20 [9%]	11 [7%]	6 [4%]	230 [18%]
45-54 years	157 [27%]	25 [15%]	2 [4%]	8 [11%]	197 [22%]
55+ years	115 [31%]	9 [41%]	1	1 [13%]	128 [31%]
**Totals**	**503 [26%]**	**61 [11%]**	**18 [6%]**	**18 [6%]**	**614 [19%]**

**ICD10 code-J: diseases of the respiratory system**					
< 35 years	27 [10%]	7 [6%]	1 [1%]	6 [6%]	42 [7%]
35-44 years	143 [20%]	26 [11%]	3 [2%]	11 [8%]	189 [14%]
45-54 years	185 [31%]	31 [19%]	9 [17%]	14 [20%]	241 [27%]
55+ years	131 [35%]	10 [46%]	1 [20%]	3 [38%]	146 [35%]
**Totals**	**486 [25%]**	**74 [14%]**	**14 [4%]**	**34 [11%]**	**618 [19%]**

**ICD10 code-K: diseases of the digestive system**					
< 35 years	47 [18%]	6 [5%]	1 [1%]	1 [1%]	56 [9%]
35-44 years	211 [29%]	21 [9%]	1 [1%]	1 [1%]	235 [18%]
45-54 years	188 [32%]	15 [9%]	1 [2%]	4 [6%]	208 [23%]
55+ years	59 [16%]	4 [18%]	0	0	63 [15%]
**Totals**	**505 [26%]**	**46 [8%]**	**3 [1%]**	**6 [2%]**	**562 [17%]**

**ICD10 code-X: within external causes of morbidity and mortality**					
< 35 years	122 [47%]	89 [72%]	95 [89%]	84 [88%]	410 [66%]
35-44 years	186 [26%]	183 [78%]	142 [92%]	122 [90%]	672 [51%]
45-54 years	92 [16%]	139 [84%]	47 [89%]	62 [89%]	354 [39%]
55+ years	22 [6%]	15 [68%]	5	8 [all]	56 [13%]
**Totals**	**422 [22%]**	**426 [78%]**	**289 [90%]**	**276 [89%]**	**1492 [46%]**

**ICD10 code-Y (includes undetermined intent): within external causes**					
< 35 years	21 [8%]	30 [24%]	11 [10%]	11 [12%]	76 [12%]
35-44 years	39 [5%]	49 [21%]	12 [8%]	13 [10%]	117 [9%]
45-54 years	24 [4%]	21 [13%]	4 [8%]	7 [10%]	58 [6%]
55+ years	10 [16%]	7 [32%]	0	0	18 [4%]
**Totals**	**94 [5%]**	**107 [20%]**	**27 [8%]**	**31 [10%]**	**269 [8%]**

Methadone-prescription-clients whose death was non-DRD were notably older, with 49% of such deaths having occurred at 45+ years of age. By contrast, those aged 45+ years at death accounted for only 187/546 (34%) of methadone-specific DRDs, 58/320 heroin-specific DRDs (18%) and 78/309 DRDs in which both heroin and methadone were implicated (25%).

Circulatory disease featured in 11% of all methadone-specific DRDs but for only 5.7% of DRDs in which heroin was implicated (36/629). Circulatory disease was mentioned for 34/187 (18%) methadone-specific DRDs at 45+ years of age but for only 12/136 (9%) heroin-implicated deaths aged 45+ years (p = 0.0232, Fisher’s exact test).

Respiratory disease was mentioned in 14% of methadone-specific DRDs and for 7.6% of DRDs in which heroin was implicated (48/629). Respiratory disease was mentioned for 41/187 (22%) methadone-specific DRDs at 45+ years of age and similarly for 27/136 (20%) heroin-implicated deaths aged 45+ years.

Digestive system disease was mentioned for 8% of all methadone-specific DRDs but for only 1.4% of DRDs in which heroin was implicated (9/629; p < 0.00001 for all ages). Table 3 also shows that digestive disease was co-present for 19/187 (10%) methadone-specific DRDs at 45+ years of age versus for 5/136 heroin-implicated DRDs aged 45+ years (4%: p = 0.0317, Fisher’s exact test).

Finally, undetermined intent was mentioned for 20% of 546 methadone-specific DRDs, which is twice as often as for DRDs in which heroin was implicated (9%, 58/629, p < 0.00001). For those aged 45+ at death, intent was undetermined for 28/187 methadone-specific DRDs (15%) and for 11/136 heroin-implicated DRDs (8%: p = 0.0828, Fisher’s exact test).

### 3.4 Quantity of Methadone prescribed by Age-group at Death and Opioid-specificity of DRD

By age-group at death for opioid-specific DRDs versus non-DRDs, Table 4 summarizes the quantity of methadone prescribed at baseline; and at the latest CHI’d prescription in the year prior to death. As explained in **Methods**, the period prescribed-for (whether 1 week or less; 2, 4 or 8 weeks; or longer) was not, in general, available electronically.

**Table 4:** Quantity of methadone prescribed at baseline and latest CHI-identified quantity in the year prior to death (latest prescriptions: N denotes the number analysed).

**Age-group at death** **(age-group’s** **% frequency within each** **sub-group of deaths)**	**Sub-grouping of all deaths**				**Total of** **3262 deaths**
	**1939** **Non-DRDs**	**546 Methadone-specific DRDs**	**320** **Heroin-specific DRDs**	**309** **Methadone +** **heroin DRDs**	
**< 35 years**	**261 (13.5%)**	**123 (22.5%)**	**107 (33.4%)**	**96 (31.1%)**	**620 (19.0%)**
Quantity of methadone prescribed: Mean (sd); 25^th^, median, 75^th^ percentile	**Baseline quantity:** 994 (972); 280, 700, 1400	**Baseline quantity:** 1232 (1189); 350, 950, 1820	**Baseline quantity:** 874 (842); 240, 700,1161	**Baseline quantity:** 1203 (964); 420, 980, 1743	**Baseline quantity:** 1033 (969); 300, 735, 1400
	**Latest in year prior to death, N = 228**: 1071 (1045); 355, 811, 1400	**Latest in year prior to death, N = 108**: 1121 (971); 412, 790, 1680	**Latest in year prior to death, N = 64**: 675 (684); 145, 420, 990	**Latest in year prior to death, N = 82**: 1078 (957); 275, 760, 1680	**Latest in year prior to death, N = 507**: 1009 (1019); 300, 770, 1400

**35-44 years**	**717 (37.0%)**	**236 (43.2%)**	**155 (48.4%)**	**135 (43.7%)**	**1318 (40.4%)**
Quantity of methadone prescribed: Mean (sd); 25^th^, median, 75^th^ percentile	**Baseline quantity:** 1216 (1133); 360, 980, 1680	**Baseline quantity:** 1686 (1596); 480,1260, 2240	**Baseline quantity:** 934 (866); 260, 665, 1400	**Baseline quantity:** 1358 (1137); 420, 980, 2160	**Baseline quantity:** 1271 (1212); 360, 980, 1890
	**Latest in year prior to death, N = 614**: 959 (894); 280, 708, 1330	**Latest in year prior to death, N = 219**: 1508 (1467); 490,1260, 1960	**Latest in year prior to death, N = 85**: 585 (824); 112, 280, 770	**Latest in year prior to death, N = 119**: 1011 (905); 270, 770, 1400	**Latest in year prior to death, N = 1094**: 1039 (1057); 280, 770, 1400

**45+ years**	**961 (49.6%)**	**187 (34.2%)**	**58 (18.1%)**	**78 (25.2%)**	**1324 (40.7%)**
Quantity of methadone prescribed: Mean (sd); 25^th^, median, 75^th^ percentile	**Baseline quantity:** 1128 (1147); 245, 735, 1680	**Baseline quantity:** 1700 (1569); 595,1400, 2240	**Baseline quantity:** 958 (999); 280, 630, 1400	**Baseline quantity:** 1147 (1027); 490, 840, 1680	**Baseline quantity:** 1208 (1215); 280, 840, 1810
	**Latest in year prior to death, N = 782**: 889 (942); 210, 560, 1260	**Latest in year prior to death, N = 171**: 1280 (1340); 500, 930, 1785	**Latest in year prior to death, N = 27**: 530 (536); 120, 330, 780	**Latest in year prior to death, N = 66**: 1154 (871); 420, 980, 1690	**Latest in year prior to death, N = 1075**: 964 (1012); 264, 644, 1400

**Totals**	**1939**	**546**	**320**	**309**	**3262**
All-ages quantity of methadone prescribed: Mean (sd); 25^th^, median, 75^th^ percentile	**Baseline quantity:** 1142 (1121); 280, 840, 1680	**Baseline quantity:** 1588 (1514); 450,1260, 2205	**Baseline quantity:** 919 (882); 263, 669, 1380	**Baseline quantity:** 1256 (1059); 420, 975, 1820	**Baseline quantity:** 1200 (1177); 308, 840, 1680
	**Latest in year prior to death, N = 1624**: 937 (941); 280, 660, 1330	**Latest in year prior to death, N = 498**: 1346 (1336); 490, 980, 1820	**Latest in year prior to death, N = 176**: 610 (735); 120, 330, 870	**Latest in year prior to death, N = 267**: 1067 (912); 299, 840, 1624	**Latest in year prior to death, N = 2676**: 1004(1023); 280, 700, 1400

**Boldface:** for major headings and totals.

Irrespective of age-group, the mean baseline quantity of prescribed methadone was higher at 1588 mg (95% CI: 1461–1715) for methadone-specific DRDs than for non-DRDs at 1142 mg (95% CI: 1092–1192); and for heroin-specific DRDs at 919 mg (95% CI: 822–1016), all confidence intervals non-overlapping.

The corresponding mean quantities of prescribed methadone in the year prior to death were lower by 15%, 18% and 34% respectively, at 1346 mg (95% CI: 1229–1463) for 498/546 (91%) methadone-specific DRDs versus 937 mg (95% CI: 891–983) for 1624/1933 (84%) non-DRDs; and 610 mg for 176/320 (55%) heroin-specific DRDs (95% CI: 501–719), all confidence intervals again non-overlapping - with each other and their baseline counterpart.

The proportion of deceased methadone-prescription-clients for whom we identified a CHI-indexed methadone prescription in the year prior to death was highest for methadone-specific DRDs (91%, 95% CI: 89%–94%), high also for non-DRDs (84%, 95% CI: 82%–85%); but just over half for heroin-specific DRDs (55%, 95% CI: 49%–60%).

For methadone-specific DRDs only, the baseline mean quantity prescribed increased with age-group at death from 1231 mg if under 35 years of age (95% CI: 1022 to 1442) through 1686 mg for those aged 35–44 years (95% CI for difference in means: 161 to 747 mg) to 1700 mg in the oldest age-group at death (95% CI for difference between oldest and youngest: 160 to 776 mg). For methadone-specific DRDs in the year prior to death, mean quantity prescribed did not differ significantly between the youngest and the oldest age-group (95% CI: -113 to 431 mg).

### 3.5 Logistic Regression Scores for Co-presence of Circulatory, Digestive or Respiratory Disease

For Scotland’s methadone-prescription-clients in 2009–2015 with a CHI-identified methadone-prescription in the year prior to death, the logistic regressions in Table 5 show that circulatory disease was more likely to be co-present for methadone-specific DRDs than for heroin-implicated DRDs; and that the odds increased dramatically with age to be 12 times higher at 55+ years (95% CI: 4–33) than if aged under 35 years at opioid-specific DRD.

**Table 5:** Logistic regression odds-ratios for co-presence of ICD10-code for circulatory; respiratory; digestive disease at 941 eligible opioid-related DRDs; and for co-presence of Y-code (undetermined intent). Interaction tested: aged 45+ years at opioid-DRD and quantity of methadone from deceased’s latest CHI-identified prescription in the year prior to opioid-DRD in top quintile.

**Covariate**	Odds Ratio coefficients (95% confidence interval)**				
	Circulatory 83 co-present (without/with interaction)		Respiratory 109 co-present	Digestive 52 co-present	Y-code 135 co-present
Risk-constant relates to baseline individual (methadone-specific DRD, aged < 35 years at death, middle three quintiles of latest methadone prescription in the year prior to death)					
Risk-constant	0.05 (0.02 to 0.10)	0.06 (0.03 to 0.12)	0.06 (0.03 to 0.11)	0.05 (0.02 to 0.12)	0.29 (0.19 to 0.43)

**Opioid specificity of 941 opioid-related DRDs** (baseline: 498 methadone-specific DRDs)					
176 Heroin-specific DRDs	0.53 (0.37 to 0.76)	0.50 (0.24 to 1.06)	0.66 (0.32 to 1.33)	0.13 (0.03 to 0.54)	0.50 (0.28 to 0.88)
267 Heroin and methadone-DRD	0.44 (0.24 to 0.82)	0.43 (0.23 to 0.81)	1.01 (0.63 to 1.62)	0.16 (0.06 to 0.44)	0.39 (0.24 to 0.64)

**Age-group at opioid-related DRD** (baseline: under 35 years of age at death)					
35-44 years	1.86 (0.90 to 3.84)	1.92 (0.93 to 3.96)	1.90 (0.97 to 3.69)	1.73 (0.16 to 19.3)	0.84 (0.54 to 1.29)
45-54 years	3.39 (1.61 to 7.13)	2.44 (1.11 to 5.33)	4.76 (2.44 to 9.27)	2.14 (0.87 to 5.27)	0.54 (0.32 to 0.91)
55+ years	11.59 (4.10 to 32.7)	8.50 (2.89 to 25.0)	15.80 (5.93 to 42.1)	4.62 (1.22 to 17.5)	0.52 (0.15 to 1.87)

**Quintile for quantity of methadone prescribed based on latest CHI-indexed prescription in the year prior to opioid-related DRD** (baseline: 557 in middle three quintiles, 245 to 1889 mg)					
Bottom: < 245 mg (n = 196)	1.55 (0.86 to 2.80)	1.51 (0.84 to 2.71)	0.79 (0.43 to 1.44)	1.30 (0.58 to 2.90)	0.64 (0.36 to 1.14)
Top: > 1890 mg (n = 188)	1.22 (0.68 to 2.20)	0.51 (0.19 to 1.36)	1.14 (0.68 to 1.90)	1.28 (0.66 to 2.50)	1.58 (1.03 to 1.46)

**Interaction: top quintile for quantity of methadone prescribed and aged 45+ years at opioid-related DRD**					
Top quintile * 45+ years at opioid-DRD		4.90 (1.45 to 16.5)			
p-value for INTERACTION		P ˜ 0.011	P ˜ 0.30	P ˜ 0.36	P ˜ 0.31
Regression chi-square (df) **with**/without INTERACTION	**46.35** (8 df) 39.20 (7 df)		**56.62** (8 df) 55.56 (7 df)	**39.05** (8 df) 38.19 (7 df)	**35.45** (8df) (7df)

**Boldface**: for covariate headings. **Please see **APPENDIX**^1^ for the corresponding Odds Ratios for 1175 opioid-related deaths in which quintile relates to the quantity of methadone prescribed according to the deceased’s latest CHI-identified methadone-prescription.

The odds on co-present respiratory disease also increased sharply with age, being 16 times higher at 55+ years (95% CI: 6–42) than if aged under 35 years at opioid-specific DRD - but opioid-specificity was not itself strongly influential.

The odds on co-present digestive disease were markedly lower for heroin-implicated DRDs than for methadone-specific DRDs; and higher if aged 55+ years at opioid-specific DRD.

Quintile for prescribed methadone was not strongly influential on the co-presence of any of the three co-morbidities of primary interest but the top quintile was (just) associated with increased odds on undetermined intent. Undetermined intent was significantly less likely in with heroin-implicated DRDs than for methadone-specific DRDs. The odds on undetermined intent was highest for those aged under 35 years.

### 3.6 Adding Interaction between Top Quintile for prescribed Methadone and Older Age at Opioid-specific DRD

The fitted interaction term assessed whether, for those aged 45+ years at the time of opioid-specific DRD, having latterly received the top quintile of prescribed methadone had was positively associated with co-presence of circulatory, digestive or respiratory disease.

Only one such interaction term, for co-presence of circulatory disease, was statistically significant (p = 0.011 before; 0.033 after Bonferroni correction). From Table 5, the estimated odds on co-present circulatory disease at methadone-specific DRD of a 55+ year old methadone-prescription-client who had received top quintile of prescribed methadone would be:

Odds without interaction: 0.05 * 1 * 11.59 * 1.22 = 0.71 and so estimated probability for co-present circulatory disease of 42%

Odds **with** interaction: 0.06 * 1 * 8.50 * 0.51 * 4.90 = 1.27 and so estimated probability for co-present circulatory disease of 56%.

For a client aged 45–54 years:

Odds without interaction: 0.05 * 1 * 3.39 * 1.22 = 0.21 and so estimated probability for co-present circulatory disease of 17%

Odds **with** interaction: 0.06 * 1 * 2.44 * 0.51 * 4.90 = 0.37 and so estimated probability for co-present circulatory disease of 27%.

See **APPENDIX**^1^ for corroborating logistic regressions for all 1175 opioid-specific DRDs using quintile for quantity of methadone prescribed in latest CHI-identified prescription (irrespective of whether latest was in the year prior to death).

## 4 Discussion

### 4.1 Main Findings

As methadone-clients aged, the non-DRD proportion of their deaths in 2009–2015 increased from 54% (se 1.4%) at 35–44 years through 65% (se 2.0%) at 45–54 years (when neoplasms, circulatory, digestive and respiratory disease were equal-top) to 89% (se 1.5%) at 55+ years (when neoplasms alone accounted for 48% of non-DRDs).

Without additional harm reduction, the underlying causes of death at ages 45–54 years in 2009–2015 are indicative of those likely in 2019–2025 for methadone-clients currently aged 35–44 years.

Second, circulatory and digestive disease were more likely to be co-present for methadone-specific DRDs than for heroin-implicated DRDs. Following Gao et al. (2016), we not only allowed for age-relatedness but also a possible interaction between older age-group at death and top quintile for the latest CHI-identified prescribed quantity of methadone. Of our three co-morbidities, significant interaction was demonstrated for circulatory disease only (after Bonferroni correction: p = 0.033).

Age-trends were steep, and broadly similar, for co-present circulatory and respiratory disease but less prominent for digestive disease until clients reached 55+ years. Differential co-presence at methadone-specific DRDs versus heroin-implicated DRDs was strongest for digestive system disease.

### 4.2 Additional Observations

Based on prior UK studies by Merrall et al. (2012) and Pierce et al. (2015b), we had anticipated reasonable power to focus on differential co-presence of circulatory, digestive and respiratory disease among the opioid-specific DRDs experienced by Scotland’s 36,606 CHI-identified methadone-clients in 2009–2015. Data-led, we observed that undetermined intent was also significantly more likely for methadone-specific DRDs than for heroin-implicated DRDs and was associated with the top quintile for prescribed methadone.

The drop-off in CHI-identified prescriptions by the year prior to death was greatest for heroin-specific DRDs (down from 320 to 176, 55% of baseline count), and least for methadone-specific DRDs (down from 546 to 498). Even at baseline, those who subsequently experienced heroin-specific DRD received a significantly lower mean quantity of methadone (919 mg, se 49 mg); and lower still in the year prior to death, when their latest quantity of prescribed methadone had reduced to a mean of 610 mg (se 55 mg).

### 4.3 Strengths and Limitations

Notwithstanding the power of Scotland’s CHI-identified methadone-prescription-client cohort for investigating co-present circulatory, digestive and respiratory system disease at 1175 opioid-specific DRDs, a major limitation was that not all methadone-prescriptions are CHI-identified. Hence, a client’s last CHI-identified methadone-prescription does not assuredly represent the client’s last methadone-prescription. Indeed, only 941/1175 (80%) of clients who experienced opioid-specific DRD had a CHI-identified methadone prescription in the year prior to death, the proportion was lowest at 55% (176/320) for heroin-specific deaths. As a precaution, our logistic regressions were repeated using quintiles of prescribed methadone at latest CHI-identified prescription (whether or not in the year prior to death), see **APPENDIX**^1^ for mostly minor differences.

Secondly, clients’ daily-dose of prescribed methadone was not routinely available in electronic format. Unfortunately, most international cohorts and also UK coroners’ records lack information on daily-dose of prescribed methadone (Caul, 2018; Degenhardt et al., 2009; Sordo et al., 2017; Xia et al., 2015). We must do better.

We did not request linkage to prescriptions for benzodiazepines, medications for chronic pain or for mental ill-health, primarily because methadone-clients’ non-prescribed access to such medications cannot be accounted for.

Co-presence of circulatory, digestive and respiratory system disease at opioid-specific DRD was ascertained at forensic post-mortem. As such, the analysed co-presences were pathological surrogates for methadone-clients’ circulatory, digestive and respiratory system co-morbidities in life, which might otherwise be captured (or not) by their ICD10 discharge-codes from hospitalizations, see Merrall et al. (2013) and Merrall (2012). We suggest that the post-mortem evidence presented here constitutes a strong argument for future linkages, in Scotland and internationally, to include clients’ ICD10-coded hospitalizations to learn about diagnosed morbidities. Notice that comorbidities for non-DRDs would generally have been determined by medical history without post-mortem evidence.

### 4.4 Other Evidence and Implications

An international meta-analysis by Sordo et al. (2017) of 19 cohort studies compared overall mortality and overdose fatalities during and after opioid substitution for around 130,000 people treated with methadone and 16,000 who received buprenorphine: substantially lower rates were reported for buprenorphine. However, due to potential confounding between choice of opioid substitution and clients’ risk-characteristics, the authors were cautious in drawing inferences about which opioid substitution to prefer. In a primary care cohort, Hickman et al. (2018) tried to adjust for confounding between choice of opioid substitution (methadone versus buprenorphine) and age-group or the client’s number of co-morbidities but were handicapped in so doing because DRDs numbered only 87. Interactions with co-morbidity or age-group were intriguing but tentative: buprenorphine was increasingly favoured when co-morbidities were present; methadone was preferred under 30 years of age but buprenorphine for new clients aged 50+ years. Hickman et al. (2018) commended switching the patient’s opioid substitution therapy to account for older age or co-morbidities. Their recommendation has support in our analysis. However, we do not under-estimate the difficulties for patients and clinicians: switching is not risk-free.

Systematic review of opioid drugs in patients with liver disease by Soleimanpour et al. (2016) noted that, in patients with reduced hepatic function (Hutchinson et al., 2005a, 2005b; Mcdonald et al., 2008), clearance of opioids decreases in liver failure and their bioavailability, including half-life, increases. The World Health Organization’s listing (World Health Organization, 2014) of people at higher risk of opioid overdose includes opioid users who have medical conditions such as HIV, liver or lung disease or suffer from depression; but fails to mention circulatory disease. Our results call for a re-think on circulatory disease.

Elsewhere we have recommended that older methadone clients (35+ years) should be offered periodic electrocardiograms if receiving a higher daily dose of methadone, for example in excess of 90 mg, see Pierce et al. (2018); and also that all methadone-clients should be prescribed take-home naloxone or naloxone-on-release (Bird et al., 2017, 2015; Parmar et al., 2016).

### 4.5 Concluding Remarks

Our results do not undermine the strong evidence-base that, while clients receive opioid substitution therapy, their risk of dying is substantially reduced (Kimber et al., 2010; Sordo et al., 2017; Hickman et al., 2018; Independent Expert Working Group on Drug Misuse and Dependence, 2017). But our findings do have actionable implications for clinical practice, namely: the major co-morbidities of ageing methadone-clients need to be mitigated; or better managed.

In particular, clients with circulatory or digestive system disease (whether HCV or alcohol related) may need their methadone dose to be reviewed if there is QTc prolongation or if there is prolongation of methadone’s half-life by liver compromise. Treatment of clients’ HCV-related liver disease by directly-acting antivirals and smoking cessation treatment can mitigate leading cancers as well as circulatory, digestive and respiratory co-morbidities.

In addition, harm reduction – such as electrocardiogram-checks, see Anchersen et al. (2009); medication-reviews, such as to reduce co-prescription of benzodiazepines with methadone (Hickman et al., 2018; Pierce et al., 2015b); and moderated alcohol intake - should be prioritized not only to reduce clients’ risk of major causes of non-DRDs but to help identify older clients whose methadone-dose may need to be reduced to below 100 mg/day or for whom a switch to buprenorphine may be considered.

Besides the increased risk of methadone-specific DRD which methadone at too-high a dose may represent for older clients with circulatory or digestive disease, our finding of substantially lower quantity of prescribed methadone in clients who subsequently experienced heroin-specific DRD reminds readers that sub-therapeutic doses of methadone risk recourse to heroin.

## Role of Funding Source

Record-linkage was funded by MRC Biostatistics Unit. The funder otherwise played no part in the decision about what to publish, when and where.

## Contributors

JRR’s long-standing interest in the mortality of ageing drug users, with increasing numbers of non drugs-related deaths (non-DRDs) as users age, led to our request for the ICD10-classification of users’ non-DRDs and for co-present morbidities at DRDs.

All three authors (LG, JRR, SMB) wanted to understand the role of circulatory, respiratory and digestive system disease in methadone-specific deaths; and interaction with the top quintile of prescribed methadone.

SMB worked out the likely power of the proposed record-linkage study on Scotland’s CHI-identified methadone-clients, 2009–2015 and drafted the Public Benefit and Privacy Panel proposal accordingly. LG, with input from SMB, analysed the data which were accessed via Scotland’s safe haven. All authors contributed to the drafting and critique of the paper.

All authors have approved the final version of the paper.

## Conflict of interest

LG has no conflicts of interest.

JRR chaired Scotland’s National Forum on Drugs-Related Deaths and now chairs Scotland’s Partnership for Action on Drugs. He was a member of the Independent Expert Working Group on Drug Misuse and Dependence (chairman: Professor Sir John Strang) which produced *Clinical Guidelines on Drug Misuse and Dependence, Update 2017.* London: Department of Health Guidelines, 2017.

SMB holds GSK shares; was a member of Scotland’s National Naloxone Advisory Group (2010-14); and was one of three co-principal investigators for the MRC-funded prison-based N-ALIVE pilot Trial of naloxone-on-release.

## Data statement

Record-linkage of Scotland’s methadone-prescriptions to mortality data, as analysed in this paper, was approved by Scotland’s Public Benefit and Privacy Panel. Analysis was conducted under safe-haven restrictions. Data access by others requires them to make application for approved access to Scotland’s Public Benefit and Privacy Panel.

## Footnotes

1 Supplementary material can be found by accessing the online version of this paper at http://dx.doi.org10.1016/j.drugalcdep.2019.01.019.
