# Supplementary material for: Non drug-related and opioid-specific causes of 3262 deaths in Scotland’s methadone-prescription clients, 2009–2015
Source: Drug Alcohol Depend. 2019 Apr 1;197:262–70. doi: 10.1016/j.drugalcdep.2019.01.019 (PMC6445802; doi:10.1016/j.drugalcdep.2019.01.019)
Supplement: Supplementary file 1 [file mmc1.docx]

**APPENDIX of Supplementary Material**

**Table A1**: ICD10 chapters which featured as co-present in methadone-prescription-clients’ 3,262 deaths

| ICD10 codes | ICD10 chapter | Frequency (%)  in 1939 non-DRDs | Frequency (%)  in 546 methadone-specific DRDs | Frequency  (%)  in 320 heroin-specific DRDs | Frequency  (%) in  309 DRDs  in which both methadone & heroin were implicated, but not buprenorphine | Frequency in 3262 deaths |
| --- | --- | --- | --- | --- | --- | --- |
| A | **Certain infectious** and parasitic diseases | 163 [ 8%] | 1 | 0 | 0 | 164 |
| B |  | **345 [18%]** | 13 [ 2%] | 0 | 1 | **360** |
| C | **Neoplasms** | **400 [21%]** | 2 | 0 | 1 | **403** |
| E | Endocrine, nutritional and metabolic diseases | 107 [ 6%] | 12 [ 2%] | 0 | 1 | 121 |
| F | **Mental and behavioural disorders** | **588 [30%]** | 498 [91%] | 307 [96%] | 293 [95%] | **1827** |
| G | Diseases of the nervous system | 91 [ 5%] | 8 | 5 | 4 | 111 |
| I | **Diseases of circulatory system** | **503 [26%]** | 61 [11%] | 18 [ 6%] | 18 [ 6%] | **614** |
| J | **Diseases of respiratory system** | **486 [25%]** | 74 [14%] | 14 [ 4%] | 34 [11%] | **618** |
| K | **Diseases of digestive system** | **505 [26%]** | 46 [ 8%] | 3 [ 1%] | 6 [ 2%] | **562** |
| R | Symptoms, signs and abnormal clinical and laboratory findings, not elsewhere classified | 216 [11%] | 24 [ 4%] | 8 [ 2%] | 4 [ 1%] | 308 |
| S | **Injury, poisoning and certain other consequences of external causes** | 115 [ 6%] | 0 | 1 | 1 | 119 |
| T |  | **463 [24%]** | 533 [98%] | 315 [98%] | 307 [99%] | **1705** |
| X | **External causes of morbidity and mortality** | **422 [22%]** | 426 [78%] | 289 [90%] | 276 [89%] | **1492** |
| Y |  | 94 [ 5%] | 107 [20%] | 27 [ 8%] | 31 [10%] | 269 |

**Boldface:** used for ICD10-chapters co-present for at least 10% of 3262 deaths

**Table A2**: ICD10 chapter for co-present causes of 1,939 non-DRDs in Scotland’s methadone-prescription cohort, 2009-2015 **by age-at-death**.

| ICD10 codes | | ICD10 chapter | Age-at-death frequency of ICD10 chapter for co-present causes of methadone-prescription-clients’ non-DRDs | | | | |
| --- | --- | --- | --- | --- | --- | --- | --- |
|  |  |  | Frequency (%) in 1939  non-DRDs | **Frequency**  **(%) in 261 clients aged**  **< 35 years**  **at death** | **Frequency**  **(%) in 717 clients aged**  **35-44 years**  **at death** | **Frequency**  **(%) in 589 clients aged**  **45-54 years**  **at death** | **Frequency**  **(%) in 372 clients aged**  **55+ years**  **at death** |
|  | | | | | | | |
| A | Certain infectious & parasitic diseases | | 163 ( 8.4%) | 19 ( 7.3%) | 75 (10.4%) | 57 ( 9.7%) | 12 ( 3.2%) |
| B |  |  | 345 (17.7%) | 20 ( 7.6%) | 132 (18.4%) | 154 (26.1%) | 39 (10.4%) |
| C | **Neoplasms** | | 400 (20.6%) | 14 ( 5.3%) | 81 (11.3%) | 114 (19.4%) | 191 (50.9%) |
| E | Endocrine, nutritional and metabolic diseases | | 107 ( 5.5%) | 13 ( 5.0%) | 44 ( 6.1%) | 32 ( 5.4%) | 18 ( 5.3%) |
| F | **Mental and behavioural disorders** | | 588 (30.3%) | 108 (41.2%) | 248 (34.6%) | 186 (31.6%) | 46 (12.3%) |
| G | Diseases of nervous system | | 91 ( 4.7%) | 14 ( 5.4%) | 38 ( 5.3%) | 28 ( 4.8%) | 11 ( 2.9%) |
| I | **Diseases of circulatory system** | | 503 (25.9%) | 41 (15.6%) | 190 (26.5%) | 157 (26.7%) | 115 (30.9%) |
| J | **Diseases of respiratory system** | | 486 (25.1%) | 27 (10.3%) | 143 (19.9%) | 185 (31.4%) | 131 (34.9%) |
| K | **Diseases of the digestive system** | | 505 (26.0%) | 47 (17.9%) | 211 (29.4%) | 188 (31.9%) | 59 (15.7%) |
| R | Symptoms, signs and abnormal clinical and laboratory findings, not elsewhere classified | | 216 (11.1%) | 28 (10.7%) | 101 (14.1%) | 66 (11.2%) | 21 ( 5.6%) |
| S | **Injury, poisoning and certain other consequences of external causes** | | 115 ( 5.9%) | 43 (16.4%) | 40 ( 5.6%) | 24 ( 4.1%) | 8 ( 2.1%) |
| T |  |  | 463 (23.9%) | 125 (47.9%) | 208 (29.0%) | 103 (17.5%) | 27 ( 7.2%) |
| X | **External causes of morbidity and mortality** | | 422 (21.8%) | 122 (46.5%) | 186 (25.9%) | 92 (15.6%) | 22 ( 5.9%) |
| Y |  |  | 94 ( 4.9%) | 21 ( 8.1%) | 39 ( 5.4%) | 24 ( 4.1%) | 10 ( 2.7%) |
| *Selected sub-codes: respiratory; digestive system; external causes* | | | | | | | |
| Influenza & pneumonia | J09-J18 | | 209 (10.8%) | 19 ( 7.3%) | 74 (10.3%) | 76 (12.9%) | 40 (10.7%) |
| Chronic lower respiratory disease | J40-J47 | | 210 (10.8%) | 4 ( 1.5%) | 51 ( 7.1%) | 85 (14.4%) | 70 (18.7%) |
|  | | | | | | | |
| Liver disease | K70-K77 | | 448 (23.0%) | 40 (15.3%) | 190 (26.5%) | 173 (29.4%) | 45 (12.0%) |
| Alcoholic liver disease | K70 | | 261 (13.5%) | 29 (11.1%) | 118 (16.5%) | 101 (17.2%) | 13 ( 3.5%) |
| Fibrosis & cirrhosis | K74 | | 91 ( 4.7%) | 4 ( 1.5%) | 28 ( 3.9%) | 42 ( 7.1%) | 17 ( 4.6%) |
|  | | | | | | | |
| Non-DRD suicide | X65-X84 &  Y15-Y34 | | 193 (10.0%) | 64 (24.4%) | 96 (13.4%) | 32 ( 5.4%) | 1 ( 0.3%) |
| Homicide | X86-Y09 | | 56 ( 2.9%) | 23 ( 8.8%) | 21 ( 2.9%) | 8 ( 1.4%) | 4 ( 1.1%) |

By ICD10 chapter and age-group at death, **Table A2** focuses on co-present causes for the methadone-prescription cohort’s 1,939 non-DRDs. Whereas 372 non-DRDs were aged 55+ years at death, only 261 non-DRDs had occurred when the client was younger than 35 years.

Three distinct age-related patterns were evident for ICD10 chapters in **Table A2**. First, the older the age-group at death, the higher was the proportion of non-DRDs with co-present cause of: neoplasms (up from 5% for non-DRDs under 35 years of age to 51% for non-DRDs at 55+ years); diseases of the circulatory system (up from 16% to 31%); diseases of the respiratory system (up from 10% to 35%). Second, by contrast, as age-group at death increased, the proportion decreased of non-DRDs with co-present cause of: mental and behavioural disorders (down from 41% for non-DRDs under 35 years of age to 12% of non-DRDs at 55+ years); injury, poisoning and certain other consequences of external causes (**S**-codes down from 16% to 2% and **T**-codes down from 48% to 7%); and external causes of morbidity and mortality (**X**-codes down from 46% to 6% and **Y**-codes down from 8% to 3%). The third pattern pertained to certain infections and parasitic diseases and to diseases of the digestive system, for both of which the associated proportion of non-DRDs appeared to be maximal at 35-44 and 45-54 years.

**Table A3**: For ICD10 code-letters I, J and K, co-present sub-codes are listed which had relative frequency of at least 2% within their respective ICD10 code-letter

| **ICD10 code-letter** [count] | Modal codes: Interpretation | Frequency |
| --- | --- | --- |
| **I**  [614, of which **342** (56%) are within the **10** selected sub-codes] | **I251 atherosclerotic heart disease** | **80** |
|  | **I259 chronic ischaemic heart disease unspecified** | **55** |
|  | **I517 cardiomegaly** | **47** |
|  | I469 cardiac arrest unspecified | 41 |
|  | I850 oesophageal varices with bleeding | 31 |
|  | I619 non-traumatic intracerebral haemorrhage, unspecified | 19 |
|  | I269 pulmonary embolism without mention of acute cor polmonale | 17 |
|  | I219 acute myocardial infarction unspecified | 15 |
|  | I279 pulmonary heart disease unspecified | 13 |
|  | *I802+I269* *phlebitis & thrombophlebitis of other deep vessels of lower extremities* | *24* |
| **J**  [618, of which **500** (81%) are within the **10** selected sub-codes] | **J180 bronchopneumonia, unspecified** | **147** |
|  | **J449 chronic obstructive pulmonary disease, unspecified** | **96** |
|  | **J189 pneumonia, unspecified** | **90** |
|  | J440 chronic obstructive pulmonary disease with acute lower respiratory infection | 39 |
|  | J690 pneumonitis due to food & vomit | 37 |
|  | J441 chronic obstructive pulmonary disease with acute exacerbation, unspecified | 36 |
|  | J448 other specified chronic obstructive pulmonary disease | 15 |
|  | J439 emphysema unspecified | 14 |
|  | J988 other specified respiratory disorders | 13 |
|  | *J449*+*J969 chronic obstructive pulmonary disease + respiratory failure, both unspecified* | *13* |
| K  [562, of which **326** (58%) are within the **7** selected sub-codes] | **K709 alcoholic liver disease unspecified** | **96** |
|  | **K746 other unspecified cirrhosis of liver** | **88** |
|  | **K703 alcoholic cirrhosis of liver** | **69** |
|  | K729 hepatic failure unspecified | 21 |
|  | K760 fatty (change of) liver, not elsewhere specified | 22 |
|  | K704 alcoholic hepatic failure | 17 |
|  | K700 alcoholic fatty liver | 13 |

**Table A4**: For ICD10 code-letters (I, J or K) or for undetermined intent (Y), quantity of methadone prescribed at baseline and in the year prior to death (latest prescription: where N denotes the number analysed).

| **Age-group at death** | **1939**  **Non-DRDs** | **546 Methadone-specific DRDs** | **320**  **Heroin-specific DRDs** | **309**  **Methadone + heroin DRDs** | **Total of 3262 deaths** |
| --- | --- | --- | --- | --- | --- |
| **ICD10 code-I: diseases of the circulatory system** [as % of the above age-group-specific deaths] | | | | | |
| **< 45 years** | **231 [24%]** | **27 [ 8%]** | **15 [ 6%]** | **9 [ 4%]** | **289 [15%]** |
| Quantity of  methadone prescribed:  Mean (sd);  25^th^, median, 75^th^ percentile | **Baseline quantity:**  1206 ( 987);  455, 910, 1820 | **Baseline quantity:**  1284 (1806);  130, 805, 1680 | **Baseline quantity:**  880 (810);  280 , 672, 1440 | **Baseline quantity:**  1199 (1227);  350, 700, 1625 | **Baseline quantity:**  1188 (1084);  360, 840, 1820 |
|  | **Year prior to death:**  **N = 207**:  1043 ( 884);  360, 840, 1512 | **Year prior to death:**  **N = 26**:  1396 (1707);  250, 773, 1540 | **Year prior to death:**  **N = 9**:  492 (469);  75, 360, 770 | **Year prior to death:**  **N = 7**:  375 ( 298);  196, 220, 770 | **Year prior to death:**  **N = 255**:  1030 ( 988);  315, 770, 1400 |
| **45+ years** | **272 [28%]** | **34 [18%]** | **3 [ 5%]** | **9 [12%]** | **325 [24%]** |
| Quantity of  methadone prescribed:  Mean (sd);  25^th^, median, 75^th^ percentile | **Baseline quantity:**  1120 (1155);  217, 717, 1680 | **Baseline quantity:**  2159 (2116);  960, 1680, 2520 | Na | **Baseline quantity:**  950 (721);  540, 560, 1400 | **Baseline quantity:**  1230 (1309);  280, 840, 1800 |
|  | **Year prior to death:**  **N = 212**:  886 ( 868);  217, 655, 1260 | **Year prior to death:**  **N = 33**:  1626 (1501);  560, 1120, 2400 |  | **Year prior to death:**  **N = 7**:  1530 ( 781);  980, 1610, 2100 | **Year prior to death:**  **N = 257**:  1001 ( 994);  280, 700, 1400 |
| **Totals** | **503 [26%]** | **61 [11%]** | **18 [ 6%]** | **18 [ 6%]** | **614 [19%]** |
| Quantity of  methadone prescribed:  Mean (sd);  25^th^, median, 75^th^ percentile | **Baseline quantity:**  1160 (1081);  294, 840, 1764 | **Baseline quantity:**  1772 (2017);  385, 1260, 2240 | **Baseline quantity:**  931 ( 799);  280, 686, 1440 | **Baseline quantity:**  1075 ( 785);  400, 630, 1400 | **Baseline quantity:**  1210 (1208);  300, 840, 1820 |
|  | **Year prior to death:**  **N = 419**:  963 ( 879);  280, 700, 1400 | **Year prior to death:**  **N = 59**:  1524 (1585);  490, 1040, 2240 | **Year prior to death:**  **N = 10**:  583 ( 527);  75, 530, 1125 | **Year prior to death:**  **N = 14**  953 ( 826);  220, 805, 1610 | **Year prior to death:**  **N = 512**:  1015 ( 990);  280, 763, 1400 |
| **ICD10 code-J: diseases of the respiratory system** [as % of the above age-group-specific deaths] | | | | | |
| **< 45 years** | **170 [17%]** | **33 [ 9%]** | **4 [ 2%]** | **17 [ 7%]** | **231 [12%]** |
| Quantity of  methadone prescribed:  Mean (sd);  25^th^, median, 75^th^ percentile | **Baseline quantity:**  1367 (1328);  420, 1120, 1820 | **Baseline quantity:**  1357 (1114);  490, 1050, 1995 | Na | **Baseline quantity:**  1620 (1045);  800, 1690, 2380 | **Baseline quantity:**  1367 (1254);  420, 1120, 1960 |
|  | **Year prior to death:**  **N = 148**:  1016 ( 948);  310, 700, 1475 | **Year prior to death:**  **N = 31**:  1675 (1906);  510, 1260, 2310 |  | **Year prior to death:**  **N = 16**:  1113 ( 987);  217, 940, 1680 | **Year prior to death:**  **N = 201**:  1114 (1167);  325, 720, 1540 |
| **45+ years** | **316 [33%]** | **41 [22%]** | **10 [17%]** | **17 [22%]** | **387 [29%]** |
| Quantity of  methadone prescribed:  Mean (sd);  25^th^, median, 75^th^ percentile | **Baseline quantity:**  1181 (1136);  280, 840, 1893 | **Baseline quantity:**  1601 (1221);  840, 1330, 2310 | **Baseline quantity:**  1327(1384);  320, 1077, 1680 | **Baseline quantity:**  1502 (1429);  700, 910, 2060 | **Baseline quantity:**  1241 (1168);  300, 980, 1960 |
|  | **Year prior to death:**  **N = 257**:  968 ( 975);  260, 700, 1400 | **Year prior to death:**  **N = 36**:  1634 (2019);  665, 1170, 2005 | **Year prior to death:**  **N = 9**:  515 ( 488);  120, 400, 630 | **Year prior to death:**  **N = 15**:  1162 (795);  420, 1040, 1736 | **Year prior to death:**  **N = 318**:  1039 (1143);  300, 777, 1400 |
| **Totals** | **486 [25%]** | **74 [14%]** | **14 [ 4%]** | **34 [14%]** | **618 [19%]** |
| Quantity of  methadone prescribed:  Mean (sd);  25^th^, median, 75^th^ percentile | **Baseline quantity:**  1247 (1209);  300, 980, 1890 | **Baseline quantity:**  1493 (1174);  550, 1260, 2205 | **Baseline quantity:**  1267 (1203);  320, 1120, 1680 | **Baseline quantity:**  1561 (1234);  700, 1207, 2240 | **Baseline quantity:**  1289 (1202);  350, 592, 1330 |
|  | **Year prior to death:**  **N = 405**:  986 ( 965);  280, 700, 1400 | **Year prior to death:**  **N = 67**:  1653 (1953);  600, 1190, 2240 | **Year prior to death:**  **N = 11**:  437 (470);  90, 280, 630 | **Year prior to death:**  **N = 31**:  1137 ( 885);  375, 1040, 1736 | **Year prior to death:**  **N = 519**:  1068 (1152);  300, 770, 1419 |
| **ICD10 code-K: diseases of the digestive system** [as % of the above age-group-specific deaths] | | | | | |
| **< 45 years** | **258 [26%]** | **27 [ 8%]** | **2 [ 1%]** | **2 [ 1%]** | **291 [15%]** |
| Quantity of  methadone prescribed:  Mean (sd);  25^th^, median, 75^th^ percentile | **Baseline quantity:**  1146 ( 974);  360, 980, 1680 | **Baseline quantity:**  1770 (1554);  450, 1750, 2100 | Na | Na | **Baseline quantity:**  1215 (1070);  385, 980, 1750 |
|  | **Year prior to death:**  **N = 223**:  910 ( 872);  210, 630, **1360** | **Year prior to death:**  **N = 27**:  1592 (1724);  400, 1400, 1840 |  |  | **Year prior to death:**  **N = 254**:  988 (1013);  240, 717, 1400 |
| **45+ years** | **247 [26%]** | **19 [10%]** | **1 [ 2%]** | **4 [ 5%]** | **271 [20%]** |
| Quantity of  methadone prescribed:  Mean (sd);  25^th^, median, 75^th^ percentile | **Baseline quantity:**  1316 (1123);  390, 1120, 1960 | **Baseline quantity:**  1607 (1297);  280, 1400, 2520 | Na | Na | **Baseline quantity:**  1337 (1131);  390, 1120, 1960 |
|  | **Year prior to death:**  **N = 195**:  886 ( 932);  280, 630, 1155 | **Year prior to death:**  **N = 19**:  1629 (1708);  520, 1040, 2100 |  |  | **Year prior to death:**  **N = 217**:  947 (1034);  280, 640, 1200 |
| **Totals** | **505 [23%]** | **46 [ 8%]** | **3 [ 1%]** | **6 [ 2%]** | **562 [15%]** |
| Quantity of  methadone prescribed:  Mean (sd);  25^th^, median, 75^th^ percentile | **Baseline quantity:**  1230 (1052);  390, 1008, 1690 | **Baseline quantity:**  1703 (1447);  450, 1715, 2412 | Na | **Baseline quantity:**  1483 ( 987);  650, 1365, 2520 | **Baseline quantity:**  1274 (1100);  390, 1050, 1890 |
|  | **Year prior to death:**  **N = 418**:  899 ( 890);  250, 630, 1260 | **Year prior to death:**  **N - 46**:  1608 (1699);  520, 1235, 1890 |  | **Year prior to death:**  **N = 4**:  750 ( 831);  231, 477, 1270 | **Year prior to death:**  **N = 471**:  969 (1022);  280, 700, 1360 |
| **ICD10 code-Y (includes undetermined intent):**  [as % of the above age-group-specific deaths] | | | | | |
| **< 45 years** | **60 [ 6%]** | **79 [22%]** | **23 [ 9%]** | **24 [10%]** | **193 [10%]** |
| Quantity of  methadone prescribed:  Mean (sd);  25^th^, median, 75^th^ percentile | **Baseline quantity:**  1194 (1134);  335, 840, 1680 | **Baseline quantity:**  2148 (1609);  700, 1960, 2800 | **Baseline quantity:**  1088 (1152);  245, 510, 1890 | **Baseline quantity:**  1655 (1164);  497, 1708, 2240 | **Baseline quantity:**  1641 (1433);  420, 1400, 2380 |
|  | **Year prior to death:**  **N = 49:**  1097 (1371);  330, 700, 1330 | **Year prior to death:**  **N = 72:**  1718 (1534);  700, 1365, 2240 | **Year prior to death:**  **N = 14:**  701 ( 765);  280, 436, 960 | **Year prior to death:**  **N = 19:**  952 ( 752);  480, 630, 1400 | **Year prior to death:**  **N = 159:**  1300 (1384);  400, 840, 1680 |
| **45+ years** | **34 [ 4%]** | **28 [15%]** | **4 [ 7%]** | **7 [ 9%]** | **76 [ 6%]** |
| Quantity of  methadone prescribed:  Mean (sd);  25^th^, median, 75^th^ percentile | **Baseline quantity:**  1148 (1097);  210, 690, 1960 | **Baseline quantity:**  2509 (2042);  623, 1837, 3920 | Na | **Baseline quantity:**  1731 (1901);  250, 840, 3720 | **Baseline quantity:**  1688 (1678);  280, 1090, 2405 |
|  | **Year prior to death:**  **N = 25:**  670 ( 797);  100, 240, 980 | **Year prior to death:**  **N = 23:**  1691 (1330);  770, 1380, 2800 |  | **Year prior to death:**  **N = 4:**  1781 (1415);  622, 1760, 2940 | **Year prior to death:**  **N = 55:**  1180 (1183);  200, 840, 1890 |
| **Totals** | **94[ 5%]** | **107 [20%]** | **27 [ 8%]** | **31 [10%]** | **269 [ 8%]** |
| Quantity of  methadone prescribed:  Mean (sd);  25^th^, median, 75^th^ percentile | **Baseline quantity:**  1177 (1115);  280, 840, 1960 | **Baseline quantity:**  2242 (1792);  665, 1960, 2940 | **Baseline quantity:**  1037 (1103);  245, 290, 1890 | **Baseline quantity:**  1672 (1328);  490, 1680, 2240 | **Baseline quantity:**  1654 (1531);  390, 1400, 2380 |
|  | **Year prior to death:**  **N = 74:**  953 (1219);  210, 532, 1120 | **Year prior to death:**  **N = 95:**  1712 (1481);  700, 1380, 2400 | **Year prior to death:**  **N = 17:**  702 ( 724);  280, 455, 960 | **Year prior to death:**  **N = 23:**  1096 ( 916);  480, 960, 1400 | **Year prior to death:**  **N = 214:**  1269 (1334);  325, 840, 1820 |

*By age-group at death for opioid-specific DRDs versus non-DRDs,* ***Table A4*** *summarizes the quantity of methadone prescribed at baseline; and for latest CHI’d prescription in the year prior to death. As explained in* ***Methods****, the period prescribed-for (whether 1 week or less; 2, 4 or 8 weeks, or longer) was not available to us.*

*Standard deviation for the quantity of methadone prescribed increases as the mean quantity increases so that logarithmic transformation is preferable when dealing with sample sizes much below 30, see* ***Table A4****.*

*Deaths with co-present circulatory disease had higher baseline and final-year mean quantity of prescribed methadone for methadone-specific DRDs than for either non-DRDs or heroin-specific DRDs but, even for methadone-specific DRDs with co-present circulatory disease, mean baseline (or latest in year prior to death) quantity of prescribed methadone was not significantly higher for clients aged 45+ years (versus < 45 years) at death.*

The mean baseline quantity of prescribed methadone was 1,772 mg for the 61 methadone-specific DRDs with co-present circulatory disease versus 11,60 mg for 503 corresponding non-DRDs (95% CI for difference in means: 97 to 1,127 mg); and 931 mg for the 18 corresponding heroin-specific DRDs (95% CI for difference in means by opioid-specificity: 214 to 1,467 mg); and also in the year prior to death. However, for methadone-specific DRDs with co-present circulatory disease, the mean quantity of methadone prescribed by age-group was significantly higher neither in the year prior to death nor at baseline (95% CI for baseline difference in mean quantity prescribed: -110 to 1,860 mg; and for final year difference: -602 to 1,062 mg).

By contrast, signals were weak for co-present respiratory disease: the mean baseline quantity of prescribed methadone was 1,493 mg for the 74 methadone-specific DRDs with co-present respiratory disease versus 1,247 mg for 486 corresponding non-DRDs (95% CI for difference in means straddled zero: -42 to 534 mg); and 1,267 mg for the 14 corresponding heroin-specific DRDs (95% CI for difference in means by opioid-specificity: -459 to 911 mg). For methadone-specific DRDs with co-present respiratory disease, the mean quantity prescribed in the final year was comparably high by age-group: 1,675 mg at under 45 years of age at death and 1,634 mg for those aged 45+ years (95% CI for difference in mean quantity prescribed in the year prior to methadone-specific DRD: -898 to 816 mg).

The mean baseline quantity of prescribed methadone was 1,703 mg for the 46 methadone-specific DRDs with co-present digestive system disease versus 1,230 mg for 505 corresponding non-DRDs (95% CI for difference in means: 45 to 901 mg). However, for methadone-specific DRDs with co-present digestive system disease, neither at baseline nor in the year prior to death did the mean quantity prescribed vary significantly with age-group: being 1,592 mg in the year prior to death for those under 45 years and 1,629 mg for those aged 45+ years at death (95% CI for difference in mean quantity prescribed in the year prior to death: -969 to 1,043 mg).

Finally, we considered deaths with co-present ICD10-code Y. Irrespective of age-group, the mean baseline quantity of prescribed methadone was 2,242 mg for the 107 methadone-specific DRDs with co-present ICD10-code Y (which includes undetermined intent) versus 1,177 mg for 94 corresponding non-DRDs (95% CI for difference in means: 657 to 1,473 mg). For methadone-specific DRDs with an associated Y-code, neither at baseline nor in the year prior to death did the mean quantity prescribed vary significantly with age-group: being 1,718 mg in their final year for those aged under 45 years at death and 1,691 mg for those aged 45+ years (95% CI for difference in mean quantity prescribed in year prior to death straddled zero: -676 to 622 mg).

**Table A5:** Logistic regression odds-ratios for co-presence of ICD10-code for circulatory; respiratory; digestive disease in all 1,175 opioid-related DRDs; and for co-presence of Y-code (undetermined intent). Interaction tested: being aged 45+ years at opioid-DRD and deceased’s last CHI-identified prescribed quantity of methadone being in top quintile.

| **Covariate** | Odds ratio coefficients (95% confidence interval) | | | | |
| --- | --- | --- | --- | --- | --- |
|  | Circulatory  97 co-present  (without/with interaction) | | Respiratory  122  co-present | Digestive  55  co-present | Y-code  165  co-present |
| Risk-constant relates to baseline individual (methadone-specific DRD, aged < 35 years at death, middle three quintiles of latest methadone prescription in the year prior to death) | | | | | |
| Risk -constant | 0.06  (0.03 to 0.10) | 0.07  (0.04 to 0.12) | 0.06  (0.03 to 0.10) | 0.04  (0.02 to 0.09) | 0.30  (0.20 to 0.43) |
| **Opioid specificity of 1175 opioid-DRDs** (baseline: 546 methadone-specific DRDs) | | | | | |
| 320 Heroin-specific DRDs | 0.55  (0.29 to 1.03) | 0.53  (0.29 to 0.95) | 0.43  (0.23 to 0.80) | 0.13  (0.04 to 0.43) | 0.40  (0.25 to 0.64) |
| 309 Heroin & methadone-DRD | 0.54  (0.31 to 0.93) | 0.52  (0.30 to 0.92) | 0.94  (0.60 to 1.48) | 0.23  (0.10 to 0.56) | 0.45  (0.29 to 0.69) |
| **Age-group at opioid-DRD** (baseline: under 35 years of age at death) | | | | | |
| 35-44 years | 1.60  (0.85 to 3.00) | 1.67  (0.89 to 3.12) | 1.77  (0.94 to 3.31) | 1.57  (0.69 to 3.57) | 0.79  (0.53 to 1.16) |
| 45-54 years | 2.77  (1.45 to 5.30) | 1.93  (0.96 to 3.92) | 4.57  (2.44 to 8.56) | 2.20  (0.95 to 5.12) | 0.53  (0.32 to 0.86) |
| 55+ years | 8.94  (7.38 to 22.0) | 6.62  (2.58 to 17.0) | 13.5  (5.57 to 32.5) | 3.67  (1.01 to 13.4) | 1.05  (0.42 to 2.64) |
| **Quintile for quantity of methadone prescribed based on latest CHI-indexed prescription prior to opioid-DRD** (baseline: 698 in middle three quintiles, 220 to 1689 mg) | | | | | |
| Bottom: < 220 mg  (n = 244) | 1.32  (0.76 to 2.29) | 1.30  (0.75 to 2.25) | 0.67  (0.28 to 0.94) | 1.23  (0.67 to 3.47) | 0.76  (0.46 to 1.23) |
| Top: > 1689 mg  (n = 233) | 1.21  (0.71 to 2.05) | 0.50  (0.20 to 1.22) | 1.30  (0.83 to 2.04) | 2.10  (1.14 to 3.85) | 1.51  (1.02 to 2.23) |
| **Interaction indicator: Top quintile of prescribed methadone on latest CHI-indexed prescription prior to death and 45+ years of age at opioid-DRD** | | | | | |
| Top quintile * 45+ years at opioid-DRD | | **5.26**  **(1.72 to 16.1)** |  | | |
| p-value for Interaction | | **P ~ 0.004** | P ~ 0.59 | P ~ 0.96 | P ~ 0.18 |
| Regression  chi-square (df) **with**/without interaction | **47.44 (8 df)**  38.01 (7 df) | | **76.98 (8 df)**  76.69 (7 df) | **46.28 (8 df)**  46.27 (7 df) | **42.20 (8 df)**  40.39 (7 df) |
